# Challenges to evaluating complex interventions: a content analysis of published papers

**DOI:** 10.1186/1471-2458-13-568

**Published:** 2013-06-11

**Authors:** Jessica Datta, Mark Petticrew

**Affiliations:** 1 Department of Social and Environmental Health Research, Faculty of Public Health and Policy, London School of Hygiene and Tropical Medicine, 15-17 Tavistock Place, WC1H 9SH, London, UK

## Abstract

**Background:**

There is continuing interest among practitioners, policymakers and researchers in the evaluation of complex interventions stemming from the need to further develop the evidence base on the effectiveness of healthcare and public health interventions, and an awareness that evaluation becomes more challenging if interventions are complex.

We undertook an analysis of published journal articles in order to identify aspects of complexity described by writers, the fields in which complex interventions are being evaluated and the challenges experienced in design, implementation and evaluation. This paper outlines the findings of this documentary analysis.

**Methods:**

The PubMed electronic database was searched for the ten year period, January 2002 to December 2011, using the term “complex intervention*” in the title and/or abstract of a paper. We extracted text from papers to a table and carried out a thematic analysis to identify authors’ descriptions of challenges faced in developing, implementing and evaluating complex interventions.

**Results:**

The search resulted in a sample of 221 papers of which full text of 216 was obtained and 207 were included in the analysis. The 207 papers broadly cover clinical, public health and methodological topics. Challenges described included the content and standardisation of interventions, the impact of the people involved (staff and patients), the organisational context of implementation, the development of outcome measures, and evaluation.

**Conclusions:**

Our analysis of these papers suggests that more detailed reporting of information on outcomes, context and intervention is required for complex interventions. Future revisions to reporting guidelines for both primary and secondary research may need to take aspects of complexity into account to enhance their value to both researchers and users of research.

## Background

There is continuing interest among practitioners, policymakers and researchers in the evaluation of complex interventions. This interest stems from the need to further develop the evidence base on the effectiveness of healthcare and public health interventions, and an awareness that evaluation becomes more challenging as interventions move along the spectrum from ‘simple’ towards more complex interventions [1]. This focus on complexity is also driven by ongoing debate about the most appropriate methods for evaluating health systems, and the recognition that it is important to know not just whether health system interventions ‘work’, but also about when, why, how and in what circumstances such interventions work well [2,3].

A further stimulus has been the Medical Research Council’s (MRC) ‘A framework for development and evaluation of RCTs for complex interventions to improve health’, originally published in 2000 [4] and revised and extended in 2008 [5]. This guidance was published in response to the difficulties faced by those attempting to develop complex interventions and evaluate their impact. It describes complex interventions as being ‘built up from a number of components, which may act both independently and inter-dependently’ [4]. These components include behaviours, behaviour parameters and methods of organising those behaviours, and they may have an effect at individual patient level, organisational or service level or population level (or all of these in some cases). The MRC’s 2008 guidance also emphasises the numbers of components and their interactions, behaviours, organisational levels and outcomes, and goes further than the 2000 framework in outlining the variability of desired outcomes and the degree to which flexibility or tailoring of the intervention is permitted. Both documents highlight the importance of establishing both *whether* an intervention is effective and *how* it works.

The term ‘complex intervention’ is now used extensively in the academic health literature to describe both health service and public health interventions. Complex interventions have been the topic of numerous conferences and meetings, the focus of funding calls, and will be the subject of a new chapter in the Cochrane Handbook for Systematic Reviews of Interventions [6]. The common usage of the term indicates increasing recognition of complexity and its implications for the development and evaluation of interventions. It may also be the case that, as the term has achieved wider application, it has come to be used strategically by researchers to add authority and currency to funding proposals and academic articles. However, it is not always clear that ‘complexity’ is being used to refer to the same things, nor what measures researchers are taking to evaluate it. It has been suggested, for example, that what is described as ‘complexity’ is actually just ‘complicatedness’ – a very different concept [7].

We undertook an analysis of published journal articles in the field of health in which complexity was an important element. Our aim was to identify the aspects of complexity described by writers; the fields in which complex interventions are being evaluated; and to describe challenges experienced due to the complexity of interventions and how authors dealt with these. This paper outlines the findings of this documentary analysis focusing, in particular, on the challenges of designing, implementing and evaluating complex interventions described by authors.

## Methods

### Search strategy

The PubMed electronic database was searched for journal articles published in the ten year period, January 2002 to December 2011. The start date was chosen to allow enough time for papers referring to the MRC guidance (2000) to have been published. The search identified the term “complex intervention*” in the title and/or abstract of a paper, excluding papers not written in English. Research reports, trial protocols, systematic reviews, meta-analyses, discussion pieces, published oral presentations and letters were included. We then undertook a content analysis of the papers to identify authors’ descriptions of challenges faced in developing, implementing and evaluating complex interventions. The search was undertaken systematically as described above but we did not conduct a critical analysis of each paper as our principal aim was to provide a snapshot of current practice rather than a comprehensive review.

### Analysis

Having read the papers, we extracted text to a table from each paper. Columns included title, author, study topic (e.g. clinical, public health, etc.), definitions of complex interventions used by authors, problems identified by authors (using the search terms: *challenge*, *barrier*, *difficult*, *limit* to identify difficulties described), and cited literature on complex interventions.

The process of analysing the papers’ content and identifying challenges described by authors produced a number of themes which we used to structure the results section. These are: intervention design (descriptions of challenges derived from the nature or content of the intervention); intervention implementation (challenges in implementing complex interventions); contextual characteristics (aspects of context that may influence implementation or evaluation of complex interventions); outcomes (reflecting the difficulties posed by the outcomes of complex interventions) and evaluation (describing challenges to evaluation). We are not suggesting that these themes are mutually exclusive. The design and content of an intervention, for example, are influenced by the context in which it will be implemented and the methodology used to evaluate it. Quotes from papers were selected to illustrate issues raised by authors.

## Results

The search resulted in a sample of 221 papers of which full text of 216 (98%) was obtained and 207 were included in the analysis. Nine papers were excluded because their subject matter was not relevant for our purposes. A small number of the papers included were published online in 2011 but in print in 2012.

The 207 papers broadly cover clinical (45%), methodological (27%), health promotion (23%) and public health (3%) topics with a small number of ‘others’ (1%). All those included in the analysis are listed in Table 1. Some papers focus on particular health conditions, such as cancer, diabetes, HIV and mental illness; some on health and social interventions, including palliative care services, complementary therapies and decision aids; and others on methodological and theoretical issues such as causality, the use of normalisation process theory, and approaches to health promotion.

**Table 1:** Papers analysed by year of publication

**2002**	
Loeb MB.** Application of the development stages of a cluster randomized trial to a framework for valuating complex health interventions.***BMC Health Serv Res* 2002 **2:**13	Methodology
Műhlhauser I, Berger, M.** Patient education-evaluation of a complex intervention.***Diabetologia* 2002 **45:**1723-1733	Health promotion
Nazareth I, Freemantle N, Duggan C, Mason J, Haines, A.** Evaluation of a complex intervention for changing professional behaviour: the Evidence Based Out Reach(EBOR)Trial.***J Health Serv Res Policy* 2002 **7:**230-238	Health promotion
Treweek SP, Glenton C, Oxman AD, Penrose A. **Computer**-**generated patient education materials: do they affect professional practice? A systematic review.** 2002 *J Am Med Inform Assoc***4:**346-358	Health promotion
**2003**	
Emery JD.** Effect of computerised evidence based guidelines.***BMJ* 2003 **326:**394	Methodology
Glazener CM, Evans JH, Peto RE.** Tricyclic and related drugs for nocturnal enuresis in children.***Cochrane Database Syst Rev* 2003 **3:**CD002117	Clinical
Lyles JS, Hodges A, Collins C, Lein C, Given CW, Given B, D'Mello D, Osborn GG, Goddeeris J, Gardiner JC, Smith RC.** Using nurse practitioners to implement an intervention in primary care for high**-**utilizing patients with medically unexplained symptoms.***Gen Hosp Psychiatry* 2003 **25:**63-73	Clinical
Omar R.** The evidence for prosthodontic treatment planning for older, partially dentate patients.***Med Princ Pract* 2003 **12 Suppl 1:**33-42	Clinical
Rollnick S.** General practitioner screening for excessive alcohol use. Paper enables open debate about a complex intervention.***BMJ* 2003 **326:**336	Health promotion
**2004**	
Byng R, Jones R.** Mental Health Link: the development and formative evaluation of a complex intervention to improve shared care for patients with long**-**term mental illness.***J Eval Clin Pract* 2004 **10:**27-36	Methodology
Conway TL, Woodruff SI, Edwards CC, Hovell MF, Klein J.** Intervention to reduce environmental tobacco smoke exposure in Latino children: null effects on hair biomarkers and parent reports.***Tob Control* 2004 **13:**90-92	Health promotion
Drescher U, Warren F, Norton K.** Towards evidence**-**based practice in medical training: making evaluations more meaningful.***Med Educ* 2004 **12:**1288-1294	Methodology
Figar S, Waisman G, De Quiros FG, Galarza C, Marchetti M, Loria GR, Camera L, Seinhart D, Camera M.** Narrowing the gap in hypertension: effectiveness of a complex antihypertensive program in the elderly.***Dis Manag* 2004 **7:**235-243	Clinical
Glazener CM, Evans JH, Peto RE.** Complex behavioural and educational interventions for nocturnal enuresis in children.***Cochrane Database Syst Rev* 2004 **1:**CD004668	Health promotion
Gülmezoglu AM, Villar J, Grimshaw J, Piaggio G, Lumbiganon P, Langer A.** Cluster randomized trial of an active, multifaceted information dissemination intervention based on The WHO Reproductive health library to change obstetric practices: methods and design issues[ISRCTN14055385]***BMC Med Res Methodol* 2004 **4:**2	Other
Hammond A, Young A, Kidao R.** A randomised controlled trial of occupational therapy for people with early rheumatoid arthritis.***Ann Rheum Dis* 2004 **63:**23-30	Clinical
Lawson H.** The logic of collaboration in education and the human services.***J Interprof Care* 2004 **18:** 225-237	Other
Paterson C, Britten N.** Acupuncture as a complex intervention: a holistic model.***J Altern Complement Med* 2004 **10:** 791-801	Clinical
Power R, Langhaug LF, Nyamurera T, Wilson D, Bassett MT, Cowan FM.** Developing complex interventions for rigorous evaluation** - **a case study from rural Zimbabwe.***Health Educ Res* 2004 **19:**570-575	Methodology
Shemilt I, Harvey I, Shepstone L, Swift L, Reading R, Mugford M, Belderson P, Norris N, Thoburn J, Robinson J.**A national evaluation of school breakfast clubs: evidence from a cluster randomized controlled trial and an observational analysis.***Child Care Health Dev* 2004 **30:** 413-427	Health promotion
Smith S, Bury, O’Leary M, Shannon W, Tynan A, Staines A, Thompson C.** The North Dublin randomized controlled trial of structured diabetes shared care.***Fam Pract* 2004 **21:** 39-45	Clinical
Whitford DL, Roberts SH, Griffin S.**Sustainability and effectiveness of comprehensive diabetes care to a district population.***Diabet Med* 2004 **21:** 1221-1228.	Clinical
**2005**	
Bonetti D, Eccles M, Johnston M, Steen N, Grimshaw J, Baker R, Walker A, Pitts N.** Guiding the design and selection of interventions to influence the implementation of evidence**-**based practice: an experimental simulation of a complex intervention trial.***Soc Sci Med* 2005 **60:**2135-2147	Methodology
Eldridge S, Spencer A, Cryer C, Parsons S, Underwood M, Feder G.** Why modelling a complex intervention is an important precursor to trial design: lessons from studying an intervention to reduce falls**-**related injuries in older people.***J Health Serv Res Policy* 2005 **10:**133-142	Methodology
Greenhalgh T, Collard A, Begum N.** Sharing stories: complex intervention for diabetes education in minority ethnic groups who do not speak English.***BMJ* 2005 **330:**628	Health promotion
Levesque L**, .**Guilbault G, Delormier T, Potvin L.**Unpacking the black box: a deconstruction of the programming approach and physical activity interventions implemented in the Kahnawake Schools Diabetes Prevention Project.***Health Promot Pract* 2005 **6:**64-71	Health promotion
Nilsson S, Spak F, Marklund B, Baigi A, Allebeck P.** Attitudes and behaviours with regards to androgenic anabolic steroids among male adolescents in a county of Sweden.***Subst Use Misuse* 2005 **40:**1-12	Other
Robinson L, Francis J, James P, Tindle N, Greenwell K, Rodgers H.** Caring for carers of people with stroke: developing a complex intervention following the Medical Research Council framework.***Clin Rehabil* 2005 **19:**560-571	Health promotion
Rowlands G, Sims J, Kerry S.** A lesson learnt: the importance of modelling in randomized controlled trials for complex interventions in primary care.***Fam Pract* 2005 **22:**132-139.	Methodology
**2006**	
Blackwood B.** Methodological issues in evaluating complex healthcare interventions.***J Adv Nurs* 2006 **54:**612-622	Methodology
Bower P, Gilbody S, Richards D, Fletcher J, Sutton A.** Collaborative care for depression in primary care. Making sense of a complex intervention: systematic review and meta**-**regression.***Br J Psychiatry* 2006 **189:**484-493	Clinical
Byrne M, Cupples ME, Smith SM, Leathem C, Corrigan M, Byrne MC, Murphy AW.** Development of a complex intervention for secondary prevention of coronary heart disease in primary care using the UK Medical Research Council framework.***Am J Manag Care* 2006 **12:**261-266	Clinical
Corrigan M, Cupples ME, Smith SM, Byrne M, Leathem CS, Clerkin P, Murphy AW.** The contribution of qualitative research in designing a complex intervention for secondary prevention of coronary heart disease in two different healthcare systems.***BMC Health Serv Res* 2006 **6:**90	Methodology
Cullen W, Stanley J, Langton D, Kelly Y, Staines, A, Bury G.** Hepatitis C infection among injecting drug users in general practice: a cluster randomised controlled trial of clinical guidelines**’ **implementation.***Br J Gen Pract* 2006 **56:**848-856	Clinical
Dornan T, Littlewood S, Margolis SA, Scherpbier A, Spencer J, Ypinazar V.** How can experience in clinical and community settings contribute to early medical education? A BEME systematic review.***Med Teach* 2006 **28:**3-18	Health promotion
Greaves CJ, Farbus L.** Effects of creative and social activity on the health and well**-**being of socially isolated older people: outcomes from a multi**-**method observational study.***J R Soc Promot Health* 2006 **126:**134-142	Public health
Heaven B, Murtagh M, Rapley T, May C, Graham R, Kaner E, Thomson R.** Patients or research subjects? A qualitative study of participation in a randomised controlled trial of a complex intervention.***Patient Educ Couns* 2006 **62:**260-270	Methodology
Holbrook AM, Thabane L, Shcherbatykh IY, O'Reilly D.** E**-**health interventions as complex interventions: improving the quality of methods of assessment.***AMIA Annu Symp Proc* 2006 **AMIA 2006 Symposium Proceedings:** 952	Methodology
Jansen YJ, Bal R, Bruijnzeels M, Foets M, Frenken R, de Bont A.** Coping with methodological dilemmas**; **about establishing the effectiveness of interventions in routine medical practice.***BMC Health Serv Res* 2006 **6:**160	Methodology
Kasper J, Kopke S, Muhlhauser I, Heesen C.**Evidence**-**based patient information about treatment of multiple sclerosis**--**a phase one study on comprehension and emotional responses.***Patient Educ Couns* 2006 **62:**56-63	Health promotion
Kunz R, Autti-Ramo I, Anttila H, Malmivaara A, Makela M.** A systematic review finds that methodological quality is better than its reputation but can be improved in physiotherapy trials in childhood cerebral palsy.***J Clin Epidemiol* 2006 **59:**1239-1248	Methodology
MacPherson H, Thorpe L, Thomas K.** Beyond needling** - **therapeutic processes in acupuncture care: a qualitative study nested within a low**-**back pain trial.***J Altern Complement Med* 2006 **12:**873-880	Clinical
Rapley T, May C, Heaven B, Murtagh M, Graham R, Kaner EF, Thomson R.** Doctor**-**patient interaction in a randomised controlled trial of decision**-**support tools.***Soc Sci Med* 2006 **62:**2267-2278	Methodology
Salas I.** Methodology for reorganization of the cervical cancer program in Chile.***Cancer Detect Prev* 2006 **30:**38-43	Public health
Sheik A, Baig K.** An audit of the use and complications of glycoprotein IIb**/**IIIa inhibitors in percutaneous coronary intervention against national UK standards.***Cardiovasc Revasc Med* 2006 **7:**237-239	Clinical
Sisk JE, Hebert PL, Horowitz CR, McLaughlin MA, Wang JJ, Chassin MR.** Effects of nurse management on the quality of heart failure care in minority communities: a randomized trial.***Ann Intern Med* 2006 **145:**273-283	Clinical
Sturt J, Hearnshaw H, Farmer A, Dale J, Eldridge S.** The Diabetes Manual trial protocol** - **a cluster randomized controlled trial of a self**-**management intervention for type 2 diabetes[ISRCTN06315411].***BMC Fam Pract* 2006 **7:**45	Health promotion
Sturt J, Taylor H, Docherty A, Dale J, Louise T.**A psychological approach to providing self**-**management education for people with type 2 diabetes: the Diabetes Manual.***BMC Fam Pract* 2006 **7:**70	Health promotion
Sturt J, Whitlock S, Hearnshaw H.** Complex intervention development for diabetes self**-**management.***J Adv Nurs* 2006 **54:**293-303	Health promotion
Treweek S, Sullivan F.** How much does pre**-**trial testing influence complex intervention trials and would more testing make any difference? An email survey.***BMC Med Res Methodol* 2006 **6:**28	Methodology
**2007**	
Allegrante JP, Peterson MG, Cornell CN, MacKenzie CR, Robbins L, Horton R, Ganz SB, Ruchlin HS, Russo PW, Paget SA, Charlson ME.** Methodological challenges of multiple**-**component intervention: lessons learned from a randomized controlled trial of functional recovery after hip fracture.***HSS J* 2007 **3:**63-70	Methodology
Armstrong N, Hearnshaw H, Powell J, Dale J.**Stakeholder perspectives on the development of a virtual clinic for diabetes care: qualitative study.***J Med Internet Res* 2007 **9:**E23	Health promotion
Ciuffreda D, Pantaleo G, Pascual M.** Effects of immunosuppressive drugs on HIV infection: implications for solid**-**organ transplantation.***Transpl Int* 2007 **8:**649-658	Clinical
Garfein RS, Swartzendruber A, Ouellet LJ, Kapadia F, Hudson SM, Thiede H, Strathdee SA, Williams IT, Bailey SL, Hagan H, Golub ET, Kerndt P, Hanson DL, Latka MH.** Methods to recruit and retain a cohort of young**-**adult injection drug users for the Third Collaborative Injection Drug Users Study**/**Drug Users Intervention Trial** (**CIDUS III**/**DUIT**).*Drug Alcohol Depend* 2007 **91 Suppl 1:**S4-17	Methodology
Green J, Jacobs B, Beecham J, Dunn G, Kroll L, Tobias C, Briskman J.** Inpatient treatment in child and adolescent psychiatry** - **a prospective study of health gain and costs.***J Child Psychol Psychiatry* 2007 **48:**1259-1267	Clinical
Hoddinott P, Pill R, Chalmers M.** Health professionals, implementation and outcomes: reflections on a complex intervention to improve breastfeeding rates in primary care.***Fam Pract* 2007 **24:**84-91	Health promotion
Lamb SE**, **Gates S, Underwood MR, Cooke MW, Ashby D, Szczepura A, Williams MA, Williamson EM, Withers EJ, Mt Isa S, Gumber A.** Managing Injuries of the Neck Trial** (**MINT**)**: design of a randomised controlled trial of treatments for whiplash associated disorders.***BMC Musculoskelet Disord* 2007 **8:**7	Clinical
MacPherson H, Schroer S.** Acupuncture as a complex intervention for depression: a consensus method to develop a standardised treatment protocol for a randomised controlled trial.***Complement Ther* Med 2007 **15:**92-100	Clinical
May CR, Mair FS, Dowrick CF, Finch TL.** Process evaluation for complex interventions in primary care: understanding trials using the normalization process model.***BMC Fam Pract* 2007 **8:**42	Methodology
Nardi R, Scanelli G, Corrao S, Iori I, Mathieu G, Cataldi Amatrian R.** Co**-**morbidity does not reflect complexity in internal medicine patients.***Eur J Intern Med* 2007 **18:**359-368	Clinical
Paul G, Smith SM, Whitford D, O'Kelly F, O'Dowd T.** Development of a complex intervention to test the effectiveness of peer support in type 2 diabetes.***BMC Health Serv Res* 2007 **7:**136	Health promotion
Paul GM, Smith SM, Whitford DL, O'Shea E, O'Kelly F, O'Dowd T.** Peer support in type 2 diabetes: a randomised controlled trial in primary care with parallel economic and qualitative analyses: pilot study and protocol.***BMC Fam Pract* 2007 **8:**45	Health promotion
Peters-Klimm F, Muller-Tasch T, Schellberg D, Gensichen J, Muth C, Herzog W, Szecsenyi J.**Rationale, design and conduct of a randomised controlled trial evaluating a primary care**-**based complex intervention to improve the quality of life of heart failure patients: HICMan** (**Heidelberg Integrated Case Management**).*BMC Cardiovasc Disord* 2007 **7:**25	Clinical
Power R, Langhaug L, Cowan F. "**But there are no snakes in the wood**"**: risk mapping as an outcome measure in evaluating complex interventions.***Sex Transm Infect* 2007 **83:**232-236	Methodology
Protheroe J, Bower P, Chew-Graham C.** The use of mixed methodology in evaluating complex interventions: identifying patient factors that moderate the effects of a decision aid.***Fam Pract* 2007 **24:**594-600	Methodology
Protopopoff N, Van Herp M, Maes P, Reid T, Baz D, D'Alessandro U, Van Bortel W, Coosemans M.** Vector control in a malaria epidemic occurring within a complex emergency situation in Burundi: a case study.***Malar J* 2007 **6:**93	Public health
Qian X, Smith H, Huang W, Zhang J, Huang Y, Garner P.** Promoting contraceptive use among unmarried female migrants in one factory in Shanghai: a pilot workplace intervention.***BMC Health Serv Res* 2007 **7:**77	Health promotion
Spillane V, Byrne MC, Byrne, M Leathem CS, O'Malley M, Cupples ME.** Monitoring treatment fidelity in a randomized controlled trial of a complex intervention.***J Adv Nurs* 2007 **60:**343-352	Methodology
Turner DE, Helliwell PS, Woodburn J.**Methodological considerations for a randomised controlled trial of podiatry care in rheumatoid arthritis: lessons from an exploratory trial.***BMC Musculoskelet Disord* 2007 **8:**109	Methodology
Wang HE, Abo BN, Lave JR, Yealy DM.** How would minimum experience standards affect the distribution of out**-**of**-**hospital endotracheal intubations?***Ann Emerg Med* 2007 **50:**246-252	Clinical
Wiener-Ogilvie S, Pinnock H, Huby G, Sheikh A, Partridge MR, Gillies J.** Do practices comply with key recommendations of the British Asthma Guideline? If not, why not?***Prim Care Respir J* 2007 **16:** 369-377	Clinical
**2008**	
Abelson JL, Khan S, Liberzon I, Erickson TM, Young EA.** Effects of perceived control and cognitive coping on endocrine stress responses to pharmacological activation.***Biol Psychiatry* 2008 **64:**701-707	Clinical
Bosch-Capblanch X, Garner P.** Primary health care supervision in developing countries.***Trop Med Int Health* 2008 **13:** 369-383	Other
Byng R, Norman I, Redfern S, Jones R.** Exposing the key functions of a complex intervention for shared care in mental health: case study of a process evaluation.***BMC Health Serv Res* 2008 **8:**274	Methodology
Carrasco MP, Salvador CH, Sagredo PG, Marquez-Montes J, Gonzalez de Mingo MA, Fragua JA, Rodriguez MC, Garcia-Olmos LM, Garcia-Lopez F, Carrero AM, Monteagudo JL.** Impact of patient**-**general practitioner short**-**messages**-**based interaction on the control of hypertension in a follow**-**up service for low**-**to**-**medium risk hypertensive patients: a randomized controlled trial.***IEEE Trans Inf Technol Biomed* 2008 **12:**780-791	Clinical
de Salis I, Tomlin Z, Toerien M, Donovan J.** Using qualitative research methods to improve recruitment to randomized controlled trials: the Quartet study.***J Health Serv Res Policy* 2008 **13 Suppl 3:**92-96	Methodology
de Salis I, Tomlin Z, Toerien M, Donovan J.** Qualitative research to improve RCT recruitment: issues arising in establishing research collaborations.***Contemp Clin Trials* 2008 **29:**663-670	Methodology
Eisenberg ML, Elliott SP, McAninch JW.** Management of restenosis after urethral stent placement.***J Urol* 2008 179**:**991-5	Clinical
Fairall LR, Bachmann MO, Zwarenstein MF, Lombard CJ, Uebel K, van Vuuren C, Steyn D, Boulle A, Bateman ED.** Streamlining tasks and roles to expand treatment and care for HIV: randomised controlled trial protocol.***Trials* 2008 **9:**21	Clinical
Fearon KC.** Cancer cachexia: developing multimodal therapy for a multidimensional problem.***Eur J Cancer* 2008 **44:**1124-32	Clinical
Hawe P, Shiell A, Riley T.** In response to Spillane V., Byrne M.C., Byrne M., Leathem C.S., O**'**Malley M.** &**Cupples M.E.(2007)Monitoring treatment fidelity in a randomized trial of a complex intervention. Journal of Advanced Nursing 60(3), 343**-**352. Important considerations for standardizing complex interventions.***J Adv Nurs* 2008 **62:**267	Methodology
Hendriks MR, Bleijlevens MH, van Haastregt JC, Bruijn FH, Diederiks JP, Mulder WJ, Ruijgrok JM, Stalenhoef PA, Crebolder HF,van Eijk JT.** A multidisciplinary fall prevention program for elderly persons: a feasibility study.***Geriatr Nurs* 2008 **29:**186-196	Health promotion
Karamanidou C, Weinman J, Horne R.** Improving haemodialysis patients**' **understanding of phosphate**-**binding medication: a pilot study of a psycho**-**educational intervention designed to change patients**' **perceptions of the problem and treatment.***Br J Health Psychol* 2008 **13:**205-214	Health promotion
Klinkhammer-Schalke M, Koller M, Ehret C, Steinger B, Ernst B, Wyatt JC, Hofstadter F, Lorenz W.** Implementing a system of quality**-**of**-**life diagnosis and therapy for breast cancer patients: results of an exploratory trial as a prerequisite for a subsequent RCT.***Br J Cancer* 2008 **99:**415-422	Clinical
Klinkhammer-Schalke M, Koller M, Wyatt JC, Steinger B, Ehret C, Ernst B, Hofstadter F, Lorenz W.** Quality of life diagnosis and therapy as complex intervention for improvement of health in breast cancer patients: delineating the conceptual, methodological, and logistic requirements(modelling).***Langenbecks Arch Surg* 2008 **393:**1-12	Methodology
MacPherson H, Nahin R, Paterson C, Cassidy CM, Lewith GT, Hammerschlag R.** Developments in acupuncture research: big**-**picture perspectives from the leading edge.***J Altern Complement Med* 2008 **14:**883-887	Methodology
MacPherson H, Thomas K.** Self**-**help advice as a process integral to traditional acupuncture care: implications for trial design.***Complement Ther Med* 2008 **16:**101-106	Health promotion
Narahari SR, Ryan TJ, Aggithaya MG, Bose KS, Prasanna KS.** Evidence**-**based approaches for the Ayurvedic traditional herbal formulations: toward an Ayurvedic CONSORT model.***J Altern Complement Med* 2008 **14:**769-776	Methodology
Panella M, Marchisio S, Gardini A, Di Stanislao F.** A cluster randomized controlled trial of a clinical pathway for hospital treatment of heart failure: study design and population.***BMC Health Serv Res* 2008 **7:**179	Clinical
Panella M, Brambilla R, Marchisio S, Di Stanislao F.** Reducing stroke in**-**hospital mortality: organized care is a complex intervention.***Stroke* 2008 **39:**e186	Clinical
Patel VH, Kirkwood BR, Pednekar, S, Araya R, King M, Chisholm D, Simon G, Weiss H.** Improving the outcomes of primary care attenders with common mental disorders in developing countries: a cluster randomized controlled trial of a collaborative stepped care intervention in Goa, India.***Trials* 2008 **9:**4	Clinical
Perkins D, Harris MF, Tan J, Christl B, Taggart J, Fanaian M.** Engaging participants in a complex intervention trial in Australian General Practice.***BMC Med Res Methodol* 2008 **8:**55	Methodology
Sampson EL, Thune-Boyle I, Kukkastenvehmas R, Jones L, Tookman A, King M, Blanchard MR.** Palliative care in advanced dementia**; **a mixed methods approach for the development of a complex intervention.***BMC Palliat Care* 2008 **7:**8	Clinical
Sifri ZC, Kim H, Lavery R, Mohr A, Livingston DH.** The impact of obesity on the outcome of emergency intubation in trauma patients.***J Trauma* 2008 **65:**396-400	Clinical
Strong V, Waters R, Hibberd C, Murray G, Wall L, Walker J, McHugh G, Walker A, Sharpe M.** Management of depression for people with cancer(SMaRT oncology 1): a randomised trial.***Lancet* 2008 **372:**40-48	Clinical
Tee A, Calzavacca P, Licari E, Goldsmith D, Bellomo R.** Bench**-**to**-**bedside review: the MET syndrome** -**the challenges of researching and adopting medical emergency teams.***Crit Care* 2008 **12:**205	Clinical
Vaessen J, Todd D.** Methodological challenges of evaluating the impact of the Global Environment Facility**'**s biodiversity program.***Eval Program Plann* 2008 **31:**231-240	Methodology
Wille N, Bettge S, Ravens-Sieberer U.** Risk and protective factors for children**'**s and adolescents**' **mental health: results of the BELLA study.***Eur Child Adolesc Psychiatry* 2008 17 **Suppl 1:**133-147	Health promotion
Wilson PM.** The UK Expert Patients Program: lessons learned and implications for cancer survivors**' **self**-**care support programs.***J Cancer Surviv* 2008 **2:**45-52	Health promotion
**2009**	
Avery AJ, Rodgers S, Cantrill JA, Armstrong S, Elliott R, Howard R, Kendrick D, Morris CJ, Murray SA, Prescott RJ, Cresswell K, Sheikh A.** Protocol for the PINCER trial: a cluster randomised trial comparing the effectiveness of a pharmacist**-**led IT**-**based intervention with simple feedback in reducing rates of clinically important errors in medicines management in general practices.***Trials* 2009 **10:**28	Health promotion
Blackberry ID, Furler JS, Young D, Best JD.**What does it cost to establish a practice**-**nurses**-**led clinical trial in general practice?***Med J Aust* 2009 **191:** 492-495	Methodology
Bovend'Eerdt TJ, Botell RE, Wade DT.** Writing SMART rehabilitation goals and achieving goal attainment scaling: a practical guide.***Clin Rehabil* 2009 **23:**352-361	Clinical
Cagle JG, Kovacs PJ.** Education: a complex and empowering social work intervention at the end of life.***Health Soc Work* 2009 **34:**17-27	Health promotion
Colombet I, Sabatier B, Gillaizeau F, Prognon P, Begue , Durieux P.** Long**-**term effects of a multifaceted intervention to encourage the choice of the oral route for proton pump inhibitors: an interrupted time**-**series analysis.***Qual Saf Health Care* 2009 **18:**232-235	Health promotion
Conboy L, Edshteyn I, Garivaltis H.** Ayurveda and Panchakarma: measuring the effects of a holistic health intervention.***ScientificWorldJournal* 2009 **9:**272-280	Clinical
Connolly M, Grimshaw J, Dodd M, Cawthorne J, Hulme T, Everitt S, Tierney S, Deaton C.** Systems and people under pressure: the discharge process in an acute hospital.***J Clin Nurs 2009***18:**4	Clinical
Donovan JL, Lane JA, Peters TJ, Brindle L, Salter E, Gillatt D, Powell P, Bollina P, Neal DE, Hamdy FC.** Development of a complex intervention improved randomization and informed consent in a randomized controlled trial.***J Clin Epidemiol* 2009 **62:**29-36	Clinical
Dumont A, Fournier P, Fraser W, Haddad S, Traore M, Diop I, Gueye M, Gaye A, Couturier F, Pasquier, JC, Beaudoin F, Lalonde A, Hatem, M, Abrahamowicz, M.** QUARITE** (**quality of care, risk management and technology in obstetrics**)**: a cluster**-**randomized trial of a multifaceted intervention to improve emergency obstetric care in Senegal and Mali.***Trials* 2009 **10:**85	Clinical
Dyas JV, Apekey T, Tilling M, Siriwardena AN.** Strategies for improving patient recruitment to focus groups in primary care: a case study reflective paper using an analytical framework.***BMC Med Res Methodol* 2009 **9:**65	Methodology
Farquhar MC, Higginson IJ, Fagan P, Booth S.** The feasibility of a single**-**blinded fast**-**track pragmatic randomised controlled trial of a complex intervention for breathlessness in advanced disease.***BMC Palliat Care* 2009 **8:**9	Clinical
Holle R, Grassel E, Ruckdaschel S, Wunder S, Mehlig H, Marx P, Pirk O, Butzlaff M, Kunz S, Lauterberg J.** Dementia care initiative in primary practice: study protocol of a cluster randomized trial on dementia management in a general practice setting.***BMC Health Serv Res* 2009 **9:**91	Clinical
King M, Jones L, McCarthy, O, Rogers M, Richardson A, Williams R, Tookman A, Nazareth I.** Development and pilot evaluation of a complex intervention to improve experienced continuity of care in patients with cancer.***Br J Cancer* 2009 **100:**274-280	Clinical
Mao J, Hou Y, Shang H, Wang H, Wang X, Zhao Y, Niu T, Cui J, Li G, Lin Q, Shi L, Jia X, Fan R, Wang B, Ruan J.** Study on the evaluation of the clinical effects of traditional chinese medicine in heart failure by complex intervention: protocol of SECETCM**-**HF.***Trials* 2009 **10:**122	Clinical
Mesotten D, Van den Berghe G.** Clinical benefits of tight glycaemic control: focus on the intensive care unit.***Best Pract Res Clin Anaesthesiol* 2009 **23:**421-429	Clinical
Murphy AW, Cupples ME, Smith SM, Byrne M, Byrne MC, Newell J.** Effect of tailored practice and patient care plans on secondary prevention of heart disease in general practice: cluster randomised controlled trial.** 2009 *BMJ***339:**b4220	Clinical
Richards D A, Hughes-Morley A, Hayes RA, Araya R, Barkham M, Bland JM, Bower P, Cape J, Chew-Graham C A, Gask L, Gilbody S, Green C, Kessler D, Lewis G, Lovell K, Manning C, Pilling S.** Collaborative Depression Trial** (**CADET**)**: multi**-**centre randomised controlled trial of collaborative care for depression** - **study protocol.***BMC Health Serv Res* 2009 **9:**188	Clinical
Rickles D.** Causality in complex interventions.***Med Health Care Philos* 2009 **12:**77-90	Methodology
Schmidt-Kraepelin C, Janssen B, Gaebel W.** Prevention of rehospitalization in schizophrenia: results of an integrated care project in Germany.***Eur Arch Psychiatry Clin Neurosci* 2009 **259 Suppl 2:**S205-12	Clinical
Schroer S, MacPherson H, Adamson J.** Designing an RCT of acupuncture for depression**--**identifying appropriate patient groups: a qualitative study.***Fam Pract* 2009 **26:** 188-195	Clinical
Seers HE, Gale N, Paterson C, Cooke HJ, Tuffrey V, Polley MJ.** Individualised and complex experiences of integrative cancer support care: combining qualitative and quantitative data.***Support Care Cancer* 2009 **17:**1159-1167	Clinical
Simon S, Higginson I J.** Evaluation of hospital palliative care teams: strengths and weaknesses of the before**-**after study design and strategies to improve it.***Palliat Med* 2009 **23:**23-28	Clinical
Simpson SA, Butler CC, Hood K, Cohen D, Dunstan F, Evans MR, Rollnick S, Moore L, Hare M, Bekkers MJ, Evans J.** Stemming the Tide of Antibiotic Resistance** (**STAR**)**: a protocol for a trial of a complex intervention addressing the** '**why**' **and** '**how**' **of appropriate antibiotic prescribing in general practice.***BMC Fam Pract* 2009 **10:**20	Clinical
Siriwardena AN, Apekey T, Tilling M, Harrison A, Dyas JV, Middleton HC, Orner R, Sach T, Dewey M, Qureshi ZM.** Effectiveness and cost**-**effectiveness of an educational intervention for practice teams to deliver problem focused therapy for insomnia: rationale and design of a pilot cluster randomised trial.***BMC Fam Pract* 2009 **10:**9	Health promotion
Suh JW, Kim SY, Park JS, Kim YS, Kang HJ, Koo BK, Kim HS.** Comparison of triple antiplatelet therapy including triflusal and conventional dual therapy in patients who underwent drug**-**eluting stent implantation.***Int Heart J* 2009 **50:**701-709	Clinical
Thompson DR, Clark AM.** Cardiac rehabilitation: into the future.***Heart* 2009 **95:**1897-1900	Clinical
Thompson EA, Relton C.** Designing clinical trials of homeopathy for menopausal symptoms: a review of the literature.***Menopause Int* 2009 **15:**31-34	Clinical
Walker J, Cassidy J, Sharpe M.** The second Symptom Management Research Trial in Oncology** (**SMaRT Oncology**-**2**)**: a randomised trial to determine the effectiveness and cost**-**effectiveness of adding a complex intervention for major depressive disorder to usual care for cancer patients.***Trials* 2009 **10:**18	Clinical
Walker J, Cassidy J, Sharpe M.** The third symptom management research trial in oncology(SMaRT oncology-3): a randomised trial to determine the efficacy of adding a complex intervention for major depressive disorder(depression care for people with lung cancer)to usual care, compared to usual care alone in patients with lung cancer.***Trials* 2009 **10:**92	Clinical
**2010**	
Ali M, Ashburn A, Bowen A, Brodie E, Corr S, Drummond A, Edmans J, Gladman, J, Kalra L, Langhorne P, Lees KR, Lincoln N, Logan P, Mead G, Patchick E, Pollock A, Pomeroy V, Sackley C, Sunnerhagen K S, van Vliet P, Walker M, Brady M.** VISTA**-**Rehab: a resource for stroke rehabilitation trials.***Int J Stroke* 2010 **5:**447-52	Methodology
Amặlinei C, Caruntu ID, Giusca, SE, Balan, RA.** Matrix metalloproteinases involvement in pathologic conditions.***Rom J Morphol Embryol* 2010 **51:**215-28	Clinical
Cameron ID.** Models of rehabilitation** - **commonalities of interventions that work and of those that do not.***Disabil Rehabil* 2010 **32:**1051-1058	Methodology
Cousins K, Connor JL, Kypri K.** Reducing alcohol**-**related harm and social disorder in a university community: a framework for evaluation.***Inj Prev* 2010 **16:**e1-6	Health promotion
Craig LE, Bernhardt J, Langhorne P, Wu O.** Early mobilization after stroke: an example of an individual patient data meta**-**analysis of a complex intervention.***Stroke* 2010 **41:**2632-2636	Clinical
de Vlaming R, Haveman-Nies A, Van't Veer P, de Groot LC.** Evaluation design for a complex intervention program targeting loneliness in non**-**institutionalized elderly Dutch people.***BMC Public Health* 2010 **10:**552	Public health
Dorresteijn JA, Kriegsman DM, Valk GD.** Complex interventions for preventing diabetic foot ulceration.***Cochrane Database Syst Rev* 2010 CD007610	Clinical
Emsley R, Dunn G, White IR.** Mediation and moderation of treatment effects in randomised controlled trials of complex interventions.***Stat Methods Med Res* 2010 **19:**237-70	Methodology
Farquhar M, Higginson IJ, Fagan P, Booth S.** Results of a pilot investigation into a complex intervention for breathlessness in advanced chronic obstructive pulmonary disease** (**COPD**)**: brief report.***Palliat Support Care* 2010 **8:**143-149	Clinical
Hill GM, Sowden JM, Lister RK, Logan, RA, Finlay AY.** Dermatology outpatient case**-**mix survey for all Welsh Trusts, 2007.***Br J Dermatol* 2010 **162:**152-158	Clinical
Hoddinott P, Britten J, Pill R.** Why do interventions work in some places and not others: a breastfeeding support group trial.***Soc Sci Med* 2010 **70:**769-778	Health promotion
Horsburgh M, Goodyear-Smith F, Bycroft J, Mahony, F, Roy D, Miller D, Donnell E.** Lessons learnt from attempting to assess the evidence base for a complex intervention introduced into New Zealand general practice.***Qual Saf Health Care* 2010 **19:**e50	Methodology
Hunter B, Segrot J.** Using a clinical pathway to support normal birth: impact on practitioner roles and working practices.***Birth* 2010 **37:**227-236	Clinical
Hutchinson C, Lange I, Kanhonou L, Filippi V, Borchert M.** Exploring the sustainability of obstetric near**-**miss case reviews: a qualitative study in the South of Benin.***Midwifery* 2010 **26:**537-543	Methodology
Jiwa M, Mitchell G, Sibbrit D, Girgis A, Burridge L.** Addressing the needs of caregivers of cancer patients in general practice: a complex intervention.***Qual Prim Care* 2010 **18:**9-16	Health promotion
Kirkegaard P, Edwards G, Hansen B, Hansen MD, Jensen MS, Lauritzen T, Risoer MB, Thomsen JL.** The RISAP**-**study: a complex intervention in risk communication and shared decision**-**making in general practice.***BMC Fam Pract* 2010 **11:**70	Health promotion
Korb-Savoldelli V, Sabatier B, Gillaizeau F, Guillemain R, Prognon P, Begue D, Durieux P.** Non**-**adherence with drug treatment after heart or lung transplantation in adults: a systematic review.***Patient Educ Couns* 2010 **81:**148-154	Clinical
Langhorne P, Lewsey JD, Jhund PS, Gillies M, Chalmers JW, Redpath A, Briggs A, Walters M, Capewell S, McMurray JJ, MacIntyre K.** Estimating the impact of stroke unit care in a whole population: an epidemiological study using routine data.***J Neurol Neurosurg* Psychiatry 2010 **81:**1301-1305	Clinical
Mayer S, Weisser M, Till H, Grafe G, Geyer C.** Congenital myelomeningocele** - **do we have to change our management?***Cerebrospinal Fluid Res* 2010 **7:**17	Clinical
McKibbon KA, Lokker, C, Wilczynski NL, Ciliska D, Dobbins M, Davis DA, Haynes RB, Straus SE.** A cross**-**sectional study of the number and frequency of terms used to refer to knowledge translation in a body of health literature in 2006: a Tower of Babel?***Implement Sci* 2010 **5:**16	Methodology
Murray, E. Treweek, S. Pope, C. MacFarlane, A. Ballini, L. Dowrick, C. Finch, T. Kennedy, A. Mair, F. O'Donnell, C. Ong, B. N. Rapley, T. Rogers, A. May, C.** Normalisation process theory: a framework for developing, evaluating and implementing complex interventions.** BMC Med 2010 **8:**63	Methodology
O'Carroll, R. Dennis, M. Johnston, M. Sudlow, C.** Improving adherence to medication in stroke survivors** (**IAMSS**)**: a randomised controlled trial: study protocol.** BMC Neurol 2010 **10:**15	Clinical
Reavley N, Livingston J, Buchbinder R, Bennell K, Stecki C, Osborne RH.** A systematic grounded approach to the development of complex interventions: the Australian Work Health Program**--**arthritis as a case study.** Soc Sci Med 2010 **70:**342-350	Health promotion
Sankaran S, Kenealy T, Adair A, Adair V, Coster H, Whitehead N, Sheridan N, Parsons M, Marshall E, Bailey L, Price C, Crombie D, Rea H.** A complex intervention to support** '**rest home**' **care: a pilot study.***N Z Med J* 2010 **123:**41-53	Health promotion
Siddiqi K, Khan A, Ahmad M, Shafiq-ur-Rehman.** An intervention to stop smoking among patients suspected of TB** - **evaluation of an integrated approach.***BMC Public Health* 2010 **10:**160	Health promotion
Sollid SJ, Lossius HM, Nakstad AR, Aven T, Soreide E.** Risk assessment of pre**-**hospital trauma airway management by anaesthesiologists using the predictive Bayesian approach.***Scand J Trauma Resusc Emerg Med* 2010 **18:**22	Clinical
Song J, Ratner ER, Wall MM, Bartels DM, Ulvestad N, Petroskas D, West M, Weber-Main AM, Grengs L, Gelberg L.** Effect of an end**-**of**-**life planning intervention on the completion of advance directives in homeless persons: a randomized trial.***Ann Intern Med* 2010 **153:**76-84	Health promotion
Thompson A, Sullivan S, Barley M, Moore L, Rogers P, Sipos A, Harrison G.** Effectiveness of a cognitive behavioural workbook for changing beliefs about antipsychotic polypharmacy: analysis from a cluster randomized controlled trial.***J Eval Clin Pract* 2010 **16:**520-528	Health promotion
Thompson EA.** Alternative and complementary therapies for the menopause: a homeopathic approach.** Maturitas 2010 **66:**350-354	Clinical
Van Herck P, Vanhaecht K, Deneckere S, Bellemans J, Panella M, Barbieri A, Sermeus W.** Key interventions and outcomes in joint arthroplasty clinical pathways: a systematic review.***J Eval Clin Pract* 2010 **16:**39-49	Clinical
Vanhaecht K, Sermeus W, Peers J, Lodewijckx C, Deneckere S, Leigheb F, Decramer M, Panella M.** The impact of care pathways for exacerbation of Chronic Obstructive Pulmonary Disease: rationale and design of a cluster randomized controlled trial.***Trials* 2010 **11:**111	Clinical
Walker J, Fairley CK, Walker SM, Gurrin L C, Gunn JM, Pirotta MV, Carter R, Hocking JS.** Computer reminders for Chlamydia screening in general practice: a randomized controlled trial.***Sex Transm Dis* 2010 **37:**445-450	Health promotion
**2011**	
Barasa EW, English M.** Economic evaluation of package of care interventions employing clinical guidelines.***Trop Med Int Health* 2011 **16:**97-104	Methodology
Barker M, Baird J, Lawrence W, Jarman M, Black C, Barnard K, Cradock S, Davies J, Margetts B Inskip H, Cooper C.** The Southampton Initiative for Health: a complex intervention to improve the diets and increase the physical activity levels of women from disadvantaged communities.***J Health Psychol* 2011 **16:**178-191	Health promotion
Begh RA, Aveyard P, Upton P, Bhopal RS, White M, Amos A, Prescott RJ, Bedi R, Barton PM, Fletcher M, Gill P, Zaidi Q, Sheikh A.** Experiences of outreach workers in promoting smoking cessation to Bangladeshi and Pakistani men: longitudinal qualitative evaluation.***BMC Public Health* 2011 **11:**452	Health promotion
Bennell KL, Egerton T, Pua YH, Abbott JH, Sims K, Buchbinder R.** Building the rationale and structure for a complex physical therapy intervention within the context of a clinical trial: a multimodal individualized treatment for patients with hip osteoarthritis.***Phys Ther* 2011 **91:**1525-1541	Clinical
Bird L, Arthur A, Cox K. "**Did the trial kill the intervention?**" **experiences from the development, implementation and evaluation of a complex intervention.***BMC Med Res Methodol* 2011 **11:**24	Clinical
Boase S, Kim Y, Craven A, Cohn S.** Involving practice nurses in primary care research: the experience of multiple and competing demands.***J Adv Nurs* 2011 **68:**590-599	Methodology
Borglin G, Gustafsson M, Krona H.** A theory**-**based educational intervention targeting nurses**' **attitudes and knowledge concerning cancer**-**related pain management: a study protocol of a quasi**-**experimental design.***BMC Health Serv Res* 2011 **11:**233	Clinical
Brady MC, Stott DJ, Norrie J, Chalmers C, St George B, Sweeney PM, Langhorne P.** Developing and evaluating the implementation of a complex intervention: using mixed methods to inform the design of a randomised controlled trial of an oral healthcare intervention after stroke.***Trial* 2011 **12:**168	Clinical
Braun SM, Wade DT, Beurskens AJ.** Use of movement imagery in neurorehabilitation: researching effects of a complex intervention.***Int J Rehabil Res* 2011 **34:**203-208	Clinical
Burr JM, Campbell MK, Campbell SE, Francis JJ, Greene A, Hernandez R, Hopkins D, McCann SK, Vale LD.** Developing the clinical components of a complex intervention for a glaucoma screening trial: a mixed methods study.***BMC Med Res Methodol* 2011 **11:**54	Clinical
Carter G, Britton B, Clover K, Rogers K, Adams C, McElduff P.** Effectiveness of QUICATOUCH: a computerised touch screen evaluation for pain and distress in ambulatory oncology patients in Newcastle, Australia.***Psycho*-*Oncology* 2011 DOI:10.1002/pon.2020	Clinical
Charles JM, Bywater T, Edwards RT.** Parenting interventions: a systematic review of the economic evidence.***Child Care Health Dev* 2011 **37:**462-474	Health promotion
Christie D, Hudson L, Mathiot A, Cole TJ, Karlsen S, Kessel A, Kinra S, Morris S, Nazareth I, Sovio U, Wong IC, Viner RM.** Assessing the efficacy of the healthy eating and lifestyle programme** (**HELP**) **compared with enhanced standard care of the obese adolescent in the community: study protocol for a randomized controlled trial.***Trials* 2011 **12:**242	Health promotion
Costantini M, Ottonelli S, Canavacci L, Pellegrini F, Beccaro M.** The effectiveness of the Liverpool care pathway in improving end of life care for dying cancer patients in hospital. A cluster randomised trial.***BMC Health Serv Res* 2011 **11:**13	Clinical
Dowding DW, Cheyne HL, Hundley V.** Complex interventions in midwifery care: reflections on the design and evaluation of an algorithm for the diagnosis of labour.***Midwifery* 2011 **27:**654-659	Methodology
Dunn G, Fowler D, Rollinson R, Freeman D, Kuipers E, Smith B, Steel C, Onwumere J, Jolley S, Garety P, Bebbington P.** Effective elements of cognitive behaviour therapy for psychosis: results of a novel type of subgroup analysis based on principal stratification.***Psychol Med* 2011 DOI: 10.1017/S0033291711001954	Clinical
Edwards ED, Mason BW, Oliver A, Powell CV.** Cohort study to test the predictability of the Melbourne criteria for activation of the medical emergency team.***Arch Dis Child* 2011 **96:**174-179	Clinical
Farquhar MC, Ewing G, Booth S.** Using mixed methods to develop and evaluate complex interventions in palliative care research.***Palliat Med* 2011 **25:**748-757	Methodology
Farquhar MC, Prevost AT, McCrone P, Higginson IJ, Gray J, Brafman-Kennedy B, Booth S.** Study protocol: Phase III single**-**blinded fast**-**track pragmatic randomised controlled trial of a complex intervention for breathlessness in advanced disease.***Trials* 2011 **12:**130	Clinical
Forster DA, Newton M, McLachlan HL, Willis K.** Exploring implementation and sustainability of models of care: can theory help?***BMC Public Health* 2011 **11** (Suppl 5)**:** S8	Methodology
Gask L, Dowrick C, Salmon P, Peters S, Morriss R.** Reattribution reconsidered: narrative review and reflections on an educational intervention for medically unexplained symptoms in primary care settings.***J Psychosom Res* 2011 **71:**325-334	Methodology
Greenhalgh T, Campbell-Richards D, Vijayaraghavan S, Collard A, Malik F, Griffin M, Morris J, Claydon A, Macfarlane F.** New models of self**-**management education for minority ethnic groups: pilot randomized trial of a story**-**sharing intervention.***J Health Serv Res Policy* 2011 **16:**28-36	Health promotion
Gustafsson L, Nugent N, Biros L.** Occupational therapy practice in hospital**-**based stroke rehabilitation?***Scand J Occup Ther* 2011 DOI**:** 10.3109/11038128	Clinical
Haire BG.** Because we can: clashes of perspective over researcher obligation in the failed PrEP trials.***Dev World Bioeth* 2011 **11:**63-74	Methodology
Hansen DG, Bergholdt SH, Holm L, Kragstrup J, Bladt T,Sondergaard J.** A complex intervention to enhance the involvement of general practitioners in cancer rehabilitation. Protocol for a randomised controlled trial and feasibility study of a multimodal intervention.***Acta Oncol* 2011 **50:**299-306	Clinical
Hirsch O, Keller H, Krones T, Donner-Banzhoff N.** Acceptance of shared decision making with reference to an electronic library of decision aids** (**arriba**-**lib**) **and its association to decision making in patients: an evaluation study.***Implement Sci* 2011 **6:**70	Health promotion
James DM.** The applicability of normalisation process theory to speech and language therapy: a review of qualitative research on a speech and language intervention.***Implement Sci* 2011 **6:**95	Methodology
Kearns A, Whitley E, Mason P, Petticrew M, Hoy C.** Material and meaningful homes: mental health impacts and psychosocial benefits of rehousing to new dwellings.** Int J Public Health 2011 56**:**597-607	Public health
Kirkevold M, Bronken BA, Martinsen R, Kvigne K.** Promoting psychosocial well**-**being following a stroke: Developing a theoretically and empirically sound complex intervention.***Int J Nurs Stud* 2011 DOI**:** 10.1016/j.ijnurstu	Health promotion
Lee MS, Choi TY,Kim JI, Kim L, Ernst E.** Acupuncture for treating attention deficit hyperactivity disorder: a systematic review and meta**-**analysis.***Chin J Integr Med* 2011 **17:**257-260	Clinical
Lossius HM, Sollid SJ, Rehn M, Lockey DJ.** Revisiting the value of pre**-**hospital tracheal intubation: an all time systematic literature review extracting the Utstein airway core variables.***Crit Care* 2011 **15:**R26	Clinical
Maratos A, Crawford MJ, Procter S.** Music therapy for depression: it seems to work, but how?***Br J Psychiatry* 2011 **199:**92-93	Clinical
Mayo NE, Scott S.** Evaluating a complex intervention with a single outcome may not be a good idea: an example from a randomised trial of stroke case management.***Age Ageing* 2011 **40:**718-724	Clinical
McLean S, Nurmatov U, Liu JL, Pagliari C, Car J,Sheikh A.** Telehealthcare for chronic obstructive pulmonary disease.***Cochrane Database Syst Rev* 2011 DOI**:** 10.1002/14651858.CD007718.pub2.	Clinical
McQuay H.** Evidence**-**based medicine: what is the evidence that it has made a difference?***Palliat Med* 2011 **25:**394-397	Methodology
Melis RJ, Teerenstra S,Olde Rikkert MG, Borm GF.** Pseudo cluster randomization: balancing the disadvantages of cluster and individual randomization.***Eval Health Prof* 2011 **34:**151-163	Methodology
Milanese S.** The use of RCT**'**s in manual therapy**-- **are we trying to fit a round peg into a square hole?***Man Ther* 2011 **16:**403-405	Methodology
Morrison JJ, Mellor A, Midwinter M, Mahoney PF, Clasper JC.** Is pre**-**hospital thoracotomy necessary in the military environment?***Injury* 2011 **42:**469-473	Clinical
Ogilvie D, Bull F, Powell J, Cooper AR, Brand C, Mutrie N, Preston J, Rutter H.** An applied ecological framework for evaluating infrastructure to promote walking and cycling: the iConnect study.***Am J Public Health* 2011 **101:**473-481	Public health
Price S, Long AF, Godfrey M, Thomas KJ.** Getting inside acupuncture trials**--**exploring intervention theory and rationale.***BMC Complement Altern Med* 2011 **11:**22	Methodology
Sampson EL, Jones L, Thune-Boyle IC, Kukkastenvehmas R, King M, Leurent B, Tookman A, Blanchard MR.** Palliative assessment and advance care planning in severe dementia: an exploratory randomized controlled trial of a complex intervention.***Palliat Med* 2011 **25:**197-209	Clinical
Schiapparelli P, Allais G, Rolando S, Airola G, Borgogno P, Terzi MG, Benedetto C.** Acupuncture in primary headache treatment.***Neurol Sci* 2011 32 **Suppl 1:**S15-8	Clinical
Schroer S, Adamson J.** Acupuncture for depression: a critique of the evidence base.***CNS Neurosci Ther* 2011 **17:** 398-410	Methodology
Senn B, Kirsch M, Sanz CC, Karlou C, Tulus K, De Leeuw J, Ringner A, Goossens GA, Cleary V.** How cancer research could benefit from the Complex Intervention Framework: students**' **experiences of the European Academy of Nursing Science summer school.***Eur J Cancer Care* (*Engl*) 2011 **20:**1-4	Methodology
Siddiqi N, Young J, House AO, Featherstone I, Hopton A, Martin C, Edwards J, Krishnan R, Peacock R, Holt R.** Stop Delirium**! **A complex intervention to prevent delirium in care homes: a mixed**-**methods feasibility study.***Age Ageing* 2011 **40:**90-98	Clinical
Wallcraft J.** The person in health care policy development.***J Eval Clin Pract* 2011 **17:**347-349	Public health
Young K, Bunn F, Trivedi D, Dickinson A.** Nutritional education for community dwelling older people: a systematic review of randomised controlled trials.***Int J Nurs Stud* 2011 **48:**751-780	Health promotion

### Use of MRC guidance

As noted above, MRC guidance on the development and evaluation of complex interventions [4,5] has been available since 2000. Without making assumptions about whether or not citation of these guidance documents or other key references reflects the content or quality of the papers listed, we report the proportions that did so. In all, 31% (n = 64) of papers cited either the 2000 or 2008 guidance or both. Nearly a quarter (23%) cited Campbell et al.’s paper, ‘Framework for design and evaluation of complex interventions to improve health’ [8] which accompanied the 2000 guidance and 12.5% (n = 26) cited Craig et al.’s paper [1] which accompanied the launch of the 2008 MRC guidance. Other highly cited papers were: Campbell et al.’s 2007 paper ‘Designing and evaluating complex interventions to improve health care’ [9] (n = 16), Hawe, Sheill and Riley’s 2004 ‘Complex interventions: how “out of control” can a randomised controlled trial be?’ [10] (n = 15) and Oakley et al.’s 2006 ‘Process evaluation in randomised controlled trials of complex interventions’ [11] (n = 12). 43% (n = 90) of papers did not cite the guidance documents or any of the above key references.

### Intervention design

Here we outline the challenges described by writers in deciding upon and standardising the content of interventions which may include a number of components.

#### The value of a theoretical understanding

The MRC guidance advises that intervention design should be based on a theoretical understanding of how an intervention causes change. Some papers focused on the development of an explanatory framework or rationale to inform intervention design and evaluation. These included, for example, one aimed at identifying and differentiating the components of two approaches to acupuncture (biomedical and traditional). These authors describe using a ‘realist review’ approach to develop an analytical framework for their review:

‘Its first step is to uncover or identify the essential or implicit theory or theories that underlie an intervention, that is, how the intervention is thought or meant to work and its expected impacts.’ [12]

Another research team described the process of developing an optimal complex physical therapy intervention for patients with hip osteoarthritis ‘in light of current knowledge and expert opinion’ given the lack of understanding about how individual components of the therapy affect the disease process [13]. In this case, the development of a theoretical framework meant collecting evidence to help understand the aetiopathogenesis and physical impairments associated with the condition.

Others explained how they used existing models and theories to inform interventions and evaluation design. Drawing on previous studies, Borglin, Gustafsson and Krona [14] describe using the Theory of Planned Behaviour to develop a series of workshops for nurses to improve pain management for cancer patients. The Normalisation Process Model [15] was used as a theoretical framework in two RCTs in maternity care and was reported to be of value in understanding organisational contexts into which new models of care are introduced [16].

‘ …the use of this theoretical model will deepen our understanding of which factors contribute to the legitimacy of an intervention and thus the likelihood that it will be sustainable.’ [16]

Even if evidence is available, it may not be possible to predict which elements of an intervention will be acceptable to health care staff and patients, have the desired effect and be sustainable. It may also be difficult to define exactly what will constitute the intervention:

‘…developing precise inclusion criteria for such complex interventions is more problematic, because by definition it is not clear *a priori* which mechanisms have to be in place in order to define an intervention as “collaborative care”.’ [17]

Nonetheless, the authors report that developing a theoretical framework early in a study enables attention to be focused on what needs to be done to plan, implement and sustain an intervention and what is less important.

#### Standardisation and treatment fidelity

Implementing any intervention in a standard format across sites is not straightforward but standardising complex, multiple treatment interventions, which may have a number of interacting components, is difficult and, some researchers argue, standardising the form of an intervention rather than its function may not be appropriate [18]. Two main challenges to standardisation were identified in these papers: on the supply side, the likelihood of variation in the delivery of services (e.g. [19]), and, on the demand side, the wide range of patients’ diagnoses, stages of disease, needs and preferences (e.g. [20]).

‘Because of heterogeneity regarding settings, experiences, training, etc. and lack of standardisation, it is very difficult to compare different HPCTs [hospital palliative care teams]; hence the need for careful definition.’ [21]

‘In the example given […] of a Computer Decision Support System, is the intervention the software or the combination of the software and the staff working in the call centre?’ [15]

Attempting to standardise an intervention to meet the needs of researchers may lead to perverse outcomes:

‘The advantage of standardisation [in acupuncture interventions] must be offset against the disadvantage that such treatments, when obviously inadequate or inappropriate, cannot be modified, as would normally occur in routine clinical practice.’ [22]

A degree of flexibility in the design and implementation of interventions was advocated by a number of writers with the aim of ensuring that interventions could be adapted to both local circumstances and to patients’ needs.

‘…it is important to retain some flexibility, allowing adaptation of the intervention to the local context and ensuring the intervention can be tailored for individual OHC [oral health care] needs.’ [23]

As well as disparity in delivery, differences in the frequency of interventions and lack of a precise definition of the start of treatment were described (e.g. [24]). The MRC guidance [5] asserts that ‘any variation in the intervention needs recording, whether or not it is intended, so that fidelity can be assessed in relation to the degree of standardisation required by the study protocol’. Replicability would be compromised by undocumented variation.

In order to record how implementation is carried out on the ground, the authors of one paper (on the topic of secondary prevention of heart disease in general practice) suggest using a range of treatment fidelity procedures to monitor the intervention and to capture the processes involved. These procedures enhance validity and reliability with the aim of ‘reducing errors in the interpretation of study outcomes and attributing outcomes directly to the effect of the intervention’ [25]. Examples described include standardised training sessions, project manager observation, quality assurance visits to practices during intervention implementation, use of a structured recall system, research nurse observation of general practitioners and practice nurses during intervention consultations and use of practice and patient care plans to document the process of intervention delivery.

### Intervention implementation

To implement an intervention one must think at an early stage about who will be responsible for what and in what setting [5]. In the case of complex interventions, there may be a number of individuals, institutions or agencies involved across several sites. After an intervention has been trialled or evaluated (and depending on the outcome), consideration should be given to its sustainability and the ease with which it can be integrated into usual service. In this section, we consider the challenges - ranging from the philosophical to the practical - identified by writers in implementing interventions.

‘Even when the concept of RRS [rapid response systems] is believed to be advantageous, the actual implementation entails overcoming a myriad of barriers: political, financial, educational, cultural, logistic, anthropological, and emotional.’ [26]

Structural and logistical obstacles may have an impact on effective implementation in the ‘real world’ where it is not always possible to control activities and outcomes.

‘Campus Watch has undergone many changes, both structural and functional, since it was introduced in 2007; its evolution has not been guided by an overarching design and modifications have occurred for reasons that have not always been well documented.’ [27]

#### Staffing issues

Those at the front line of ‘delivering’ an intervention may face time and resource difficulties or lack of buy-in with the aims of the intervention while there may be political and/or financial considerations further up the organisational hierarchy. The replication, regulation and sustainability of new practices in diverse teams across a number of sites can make heavy demands on staff who may experience competing priorities if they are also involved in data collection for evaluation purposes:

‘There was no systematic exploration of midwives’ views of working in the models post RCT, or of the views of other stakeholders such as non-team midwives, managers and obstetric staff during or after completion of the team RCT, nor during the subsequent iterations of the team model. Therefore it is not possible to draw conclusions about why the original evaluated model was not sustained.’ [16]

‘In …the area where breastfeeding rates did not improve, health professional support for the project was weaker and relationships between midwives and health visitors were problematic.’ [28]

‘It is clear that teachers found it difficult to deliver the programme for a variety of logistic reasons (low morale, lack of support and competing priorities at school) and contextual reasons (difficulty teaching about sensitive issues, switching from their traditional teacher role, and lack of trust between pupils and teachers).’ [29]

Implementing an intervention uniformly may create difficulties for clinicians whose first aim is to provide the most effective care to patients. The papers present examples of treatment that deviated from the protocol because of decisions made by staff:

‘At least two control patients are known to have received more intensive physical therapy, i.e. muscle-strength training, than they would have otherwise. We believe that once the surgeons sensed that patients receiving intensive physical therapy were responding well, the surgeons were likely to have encouraged their patients to get more physical therapy, thus further diluting the impact of the intervention.’ [30]

#### Patient issues

A number of issues relating to patients were raised by authors. These included patients’ preferences and patient/staff interaction, and recruitment and retention to trials. Studies about the treatment of chronic illness, for example, emphasised the role of patients (and carers) in active management of health conditions [31,32] Less positively, one paper reported that for a number of reasons ‘despite initial willingness, after a few weeks some patients [suffering from psychosis] no longer wanted to receive therapy' [33]. A review on the topic of patients with medically unexplained symptoms reported that patients distrusted doctors regarding emotional aspects of their problems while doctors were concerned about encouraging patient dependence [34].

Those conducting trials reported that recruitment and retention of participants may be negatively affected if the intervention targets patients who are severely ill or who are hard to reach. Examples reported included patients with advanced dementia and their carers [35], those receiving palliative care [21] and young drug users [36]. In the first example, unbiased comparisons could not be made between intervention and control groups because of sample attrition [35]. In their consideration of the strengths and weaknesses of a before-after study design, Simon and Higginson [21] offer suggestions for strategies (including inclusion of a control group in research design, time series approaches, and more robust outcome measures) to control and limit secular trends, bias and confounders. Garfein and colleagues [36] describe one method used to retain participants:

‘Given the anticipated difficulty in retaining young IDUs [intravenous drug users]for a longitudinal study, follow-up window periods were designed such that the need for high retention was balanced with the need for uniform intervals between the intervention and follow-up assessments.’

In evaluating an intervention aimed at high-utilising patients with medically unexplained symptoms, Lyles and colleagues [37] describe how they achieved their impressive retention rate of 98%:

‘Remunerating participants in recognition of their time commitment helped to maintain interest. However, consistent, clear communication from project staff and persistence in contacting participants were also important factors in enrolling and retaining subjects. We maintained a communication link with participants at intervals throughout the project.’

### Contextual characteristics

Complex interventions, by their nature, are more likely than simpler ones to depend for their success on the context in which they are implemented [38]. Authors described the impact of structural, capacity, professional and political factors on their introduction. The most commonly cited contextual barrier to implementation was the organisational context. As one author put it:

‘The findings concur with previous studies, which suggest that organisational environment and culture, and client factors may influence occupational therapy practice.’ [39].

Organisational context encompassed a wide range of elements from the parochial to the regional or national level and included organisational cultures, such as hierarchies and professional boundaries, staffing arrangements, social, geographical and environmental barriers, and the impact of other simultaneous organisational changes. The organisational context could either help or hinder the implementation of an intervention – or do both at the same time.

‘More attention should be given to the systems into which policies and complex interventions intervene. Particularly how the negative consequences of the environment, resource shortages, organisational change, competing demands and leadership affect an organisation’s ability to effectively deliver an intervention.’ [40]

‘The difficulties of delivering complex interventions in inner city areas are well known to clinicians, and might be attributed variously to low levels of social support, high levels of deprivation, and relative residential instability. Such contextual disadvantages remain a therapeutic challenge.’ [33]

‘Although the changing of long-term entrenched practices of physicians and other professionals is known to be a difficult task, problem solving in expanding cycles was able to affect such a change and produce an effective cervical cancer screening programme with no increase in financial resources.’ [41]

Another example of an organisational barrier to implementation was lack of support for what were seen as demanding projects. GP practice staff, for example, were thought to have few incentives for engaging in thinking through and developing complex new service arrangements:

‘Furthermore the external environment was not a sufficiently supportive context for the scope of the proposed shared care developments: it was seen as “*a big project*”.’[42]

### Outcomes

Having established what outcome(s) an intervention is aiming to achieve, researchers face challenges in designing tools to effectively measure outcomes, understanding ‘the length and complexity of the causal chains linking intervention with outcome’ [5], explaining discrepancies between expected and observed outcomes, and capturing the long term characteristics of outcomes after a trial or study is concluded.

#### Multidimensional outcome measures

Outcomes are likely to be plural and multi-dimensional, spanning ‘the spectrum from mortality, morbidity, disability, to satisfaction and cost’ [43] as ‘restricting the success indicator to one single health or behavioural outcome leads to many unsolved questions about the success factors for, and barriers to, the effectiveness of the intervention’ [44]. Clinical pathways are aspects of complex interventions that may demand outcomes be measured across many domains including clinical, service, team, process and cost [38]. As well as breadth, outcome measures must take time into account and may be designed for the short, medium or long term or all three.

‘Given this degree of complexity identifying a single primary outcome measure to capture the impact of an OHC [oral health care] intervention is problematic. We would anticipate that a multifaceted OHC intervention would impact upon a range of components including for example dental referrals, staff knowledge and patients’ oral health.’ [23]

‘It is therefore critical that the impact of new models of care are rigorously evaluated, considering outcomes for women and infants as well as outcomes for midwives and other maternity care providers.’ [16]

#### Assessing outcomes

Apart from difficulties in deciding upon measureable outcomes imposed by the complexity of interventions, writers noted that there is now an expectation that the bio-psycho-social aspects of interventions be measured as well as the clinical ones [45]. In palliative care, for example, patient experience *is* the primary outcome [46]. In general, it is argued that patient-centred outcomes, such as quality of life, as well as the views and experiences of staff should be taken into account. Some authors suggested that methods of measuring outcomes did not always capture the positive impact of an intervention and, in some cases, described their use of qualitative data to measure patient experience (e.g. [47]).

‘The lack of an objective outcome was in contrast to subjective feedback from the study participants who felt that the intervention had produced a change in practice.’ [48]

‘Reliance on empirical and societal defined outcomes often hides success in terms of participant defined outcomes.’ [19]

Establishing ‘hard’ outcome measures was seen to be difficult in particular fields where the success of an intervention does not necessarily equate with patient improvement or survival.

‘The holistic approach of palliative care and its services causes some problems in defining clear outcomes and finding valid measurements.’ [21]

‘There is a lack of an accepted primary outcome regarding the use of decision aids. Possible categories to classify measures of effectiveness are knowledge, decision process (e.g., satisfaction and participation preference), decision outcomes (e.g., has a treatment decision been made, adherence), health status, and economic measures.’[49]

Some writers admitted that it was not possible to attribute the ‘active ingredient’ [4] of a complex intervention to a particular component of its design:

‘If this complex intervention does reduce mortality the relative contributions of education, PEWS [paediatric early warning system] and MET [medical emergency team] to clinical effectiveness is unknown.’ [50]

‘In many cases, the effectiveness of training is more difficult to measure because a wide range of variables unrelated to the training intervention can mediate both the training process and the outcome. These variables need to be considered if it is to be established whether an outcome is due to the training intervention or other unrelated factors. For instance, variables related to the individual have been shown to mediate impact on outcomes like stress and burnout levels, and staff satisfaction.’ [51]

### Evaluation

The process of evaluating health service interventions occurs before, during and after implementation. In this section, we highlight some important issues raised by authors but do not systematically describe the many research designs which are the subject of the papers themselves.

#### Formative and process evaluation

The 2008 MRC guidance suggests that ‘A mixture of qualitative and quantitative methods is likely to be needed, for example to understand barriers to participation and to estimate response rates’ [5] to assess the feasibility of an intervention. As noted above, qualitative data are increasingly recognised as ‘an essential component of health services research’ [52], providing insights into the acceptability of interventions and their social consequences which cannot be measured by quantitative approaches. Formative evaluation – conducted to aid intervention design – can offer insights into the views and priorities of both patients and practitioners.

‘The key to the successful development of the complex intervention was the use of qualitative research that ensured that the intervention was based on data from interactions in ongoing trial recruitment appointments. Exploratory qualitative research of recruitment appointments in the Protect feasibility study showed that improvements to the presentation of study information increased rates of randomization from 30% to over 65%.’ [53]

Process evaluation is particularly important in multisite trials, ‘where the “same” intervention may be implemented and received in different ways’ [11].

‘Neither quantitative nor qualitative approaches alone would provide an adequate insight into the implementation of the intervention across all three levels of care, from the perspective of all involved and capture the information needed in relation to both effectiveness and feasibility issues.’ [23]

## Discussion

### Limitations

The number and range of papers discussed here are not comprehensive given the search terms used and database searched and selection bias is therefore possible. However, we feel that there is a large enough number included for our purposes. We conducted a content analysis rather than a systematic review which supported our aim of identifying aspects of complexity in health interventions, the fields in which they are implemented and the challenges experiences by researchers.

### Summary of results

The literature on complex interventions is thick with descriptions of complex, challenging interventions, but thin on practical advice on how these should be dealt with. In the papers we surveyed, authors pointed to the practical value of theory in determining which features of an intervention and its context are likely to be important in influencing outcomes and determining sustainability. They caution against attempting to too narrowly define and standardise the intervention, drawing on Hawe and colleagues lead (standardising on ‘function’ rather than ‘form’) [10]. This also means having procedures in place to document what is actually done under the heading of ‘the intervention’.

The interaction between intervention and context is frequently emphasised, and one aspect of context which is highlighted in several papers is the people involved, including staff and patients themselves. The MRC guidance notes that complexity may derive from interaction between patient or recipient and provider. The implication for implementation and evaluation is that (in the case of healthcare interventions) barriers at both levels should be considered and mitigated and, in the case of evaluation, relevant data collected. These barriers could also be built into the initial logic model driving the evaluation [54]. The papers also point to the wide range of contexts which have been considered as relevant, including professional boundaries and hierarchies, which do not often feature in descriptions of context, but are clearly relevant in some of these examples. In one study the specific recommendation is made that attention should be given to the systems into which complex interventions are placed [40]. In practical terms this may mean describing those systems in detail and at different levels and theorising on how they may affect the effectiveness of implementation.

Several studies point to a multiplicity of health and non-health outcomes as a source of complexity. In many of the papers which raise this as an issue, there is an implicit need for outcome measures – or a range of outcome approaches – capable of capturing outcomes across different dimensions and time scales. This may imply a move away from a focus on primary outcomes and a small number of secondary outcomes towards a much more multi-criteria form of assessment which acknowledges the multiple objectives of many complex interventions.

## Conclusions

### Implications

The above comments may have implications for reporting of studies of complex interventions. The quotes suggest that more detailed reporting of information on outcomes, context and intervention is required for complex interventions. However, reporting guidelines for quantitative studies may require further adaptation to enable adequate explanation of complex interventions, and the contexts within which they were implemented. Defining and describing context, for example, may prove particularly challenging and, given the inherent flexibility in complex interventions themselves, even defining the intervention may be difficult. Future revisions to reporting guidelines for both primary and secondary research may need to take aspects of complexity into account to enhance their value to both researchers and users of research.

## Competing interests

The authors declare that they have no competing interests.

## Authors’ contributions

MP conceived the idea for the paper. MP and JD developed the methodology. JD carried out the search and content analysis and wrote the methodology and results section. The introduction was written by MP and JD and the discussion and conclusions by MP. Both authors read and approved the final manuscript.

## Pre-publication history

The pre-publication history for this paper can be accessed here:

http://www.biomedcentral.com/1471-2458/13/568/prepub
